# Supplementary material for: Transcriptome Analysis of Macrophytes’ Myriophyllum spicatum Response to Ammonium Nitrogen Stress Using the Whole Plant Individual
Source: Plants (Basel). 2023 Nov 16;12(22):3875. doi: 10.3390/plants12223875 (PMC10675724; doi:10.3390/plants12223875)

Supplementary

Figure S1. A comparison summary of regulations of the top 10 genes in above and below-ground parts of the macrophyte. The letters represent the genes; Serine/threonine-protein kinase STY46(STY46), Aspartate aminotransferase (AATC), L-asparaginase (ASPGB), Alpha Carbonic anhydrase (*α* CA), Ammonium transporter 1 member 2 (AMT12), Asparagine synthetase (ASNS), Glutamate synthase 1 [NADH]) (GLT1), Ferredoxin-dependent glutamate synthase 1(GLU1), NADP-specific glutamate dehydrogenase (DHE4), and Glutamine synthetase cytosolic isozyme 1(GLNA1) It was plotted using the data of the highest ammonium concentrations only (50 mg/L for leaf and stem; 1250 mg/L for Root) The red and blue represent upregulated and downregulated genes respectively.

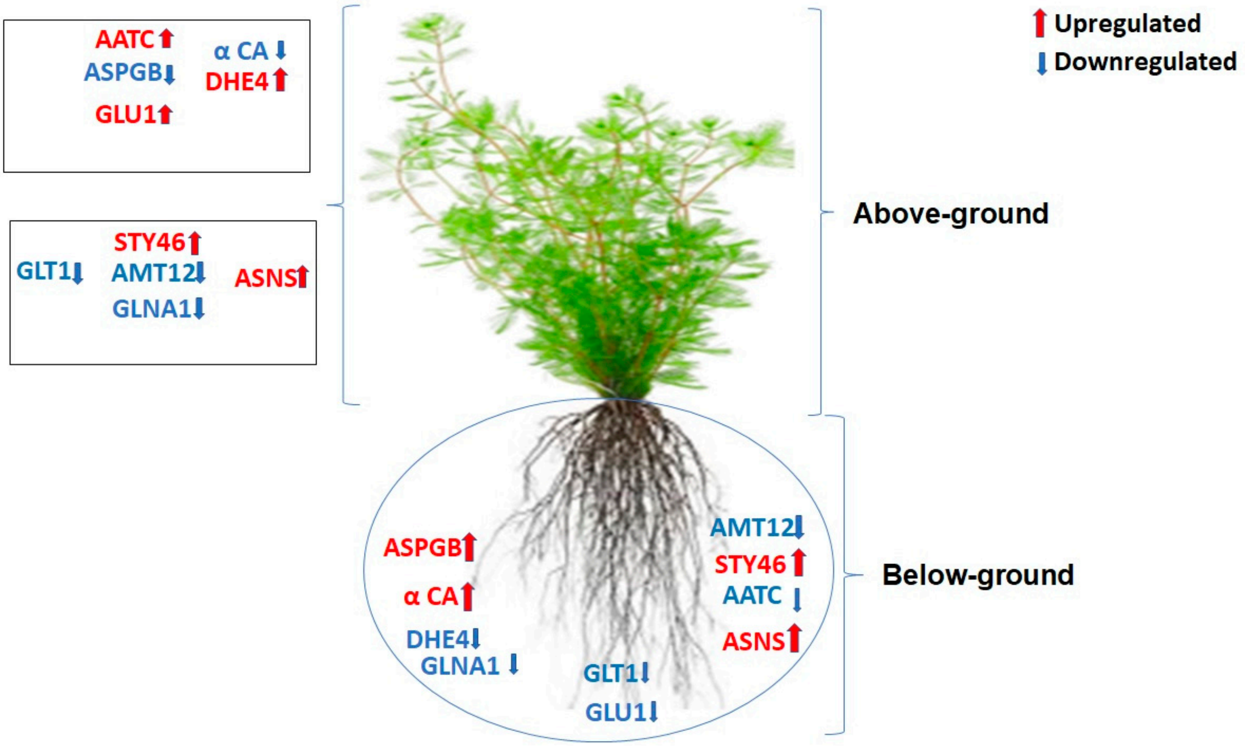

Supplement: Supplementary file 1 [file plants-12-03875-s001.zip › plants-2698111-supplementary.pdf]
